# A PU.1 Suppressive Target Gene, Metallothionein 1G, Inhibits Retinoic Acid-Induced NB4 Cell Differentiation

**DOI:** 10.1371/journal.pone.0103282

**Published:** 2014-07-29

**Authors:** Naomi Hirako, Hiroko Nakano, Shinichiro Takahashi

**Affiliations:** 1 Division of Molecular Hematology, Kitasato University Graduate School of Medical Sciences, Minami-ku, Sagamihara, Japan; 2 Division of Hematology, Kitasato University School of Allied Health Sciences, Minami-ku, Sagamihara, Japan; Western University, Canada

## Abstract

We recently revealed that myeloid master regulator *SPI1*/PU.1 directly represses metallothionein *(MT) 1G* through its epigenetic activity of PU.1, but the functions of MT1G in myeloid differentiation remain unknown. To clarify this, we established MT1G-overexpressing acute promyelocytic leukemia NB4 (NB4MTOE) cells, and investigated whether MT1G functionally contributes to all-trans retinoic acid (ATRA)-induced NB4 cell differentiation. Real-time PCR analyses demonstrated that the inductions of *CD11b* and *CD11c* and reductions in myeloperoxidase and *c-myc* by ATRA were significantly attenuated in NB4MTOE cells. Morphological examination revealed that the percentages of differentiated cells induced by ATRA were reduced in NB4MTOE cells. Since G1 arrest is a hallmark of ATRA-induced NB4 cell differentiation, we observed a decrease in G1 accumulation, as well as decreases in p21^WAF1/CIP1^ and cyclin D1 inductions, by ATRA in NB4MTOE cells. Nitroblue tetrazolium (NBT) reduction assays revealed that the proportions of NBT-positive cells were decreased in NB4MTOE cells in the presence of ATRA. Microarray analyses showed that the changes in expression of several myeloid differentiation-related genes (*GATA2*, azurocidin 1, pyrroline-5-carboxylate reductase 1, matrix metallopeptidase -8, S100 calcium-binding protein A12, neutrophil cytosolic factor 2 and oncostatin M) induced by ATRA were disturbed in NB4MTOE cells. Collectively, overexpression of MT1G inhibits the proper differentiation of myeloid cells.

## Introduction

Metallothionein (MT) proteins comprise a group of cysteine-rich low-molecular-weight intracellular proteins that are classified into four groups [1], [2]. MT1 and MT2 are the two major isoforms found in all types of tissues [3], [4]. Two other members, MT3 and MT4, are expressed in limited types of tissues [5], [6]. There are several isoforms of MT1. The known functional MT1 isoforms are MT1A, 1B, 1E, 1F, 1G, 1H, and 1X [2]. MT1G is one of the major isoforms of MT, and its mRNA is abundantly expressed in various cell types [7], [8]. The *MT* genes located in a cluster on chromosome 16 can be activated by a variety of stimuli, and the expression and induction of their encoded proteins are associated with protection against DNA damage, oxidative stress, and apoptosis [2]. The protective role of MT against oxidative stress and metal toxicity [1], [2] suggests that MT may play a role in tumor cell survival and growth. A number of studies have shown that increased MT expression is closely associated with tumor grade and proliferative activity in solid tumors [1], [2]. Compared with other tumors, however, studies on MT in hematological malignancies are relatively scarce.

PU.1 is a hematopoietic transcription factor, encoded by the *SPI1* gene, expressed in granulocytic, monocytic, and B-lymphoid cells [9]. *SPI1*-deficient mice exhibit defects in the development of neutrophils, macrophages, and B cells [10]. Mice carrying hypomorphic *SPI1* alleles that reduce PU.1 expression to 20% of its normal levels exhibit blockade of myeloid differentiation, leading to the development of acute myeloid leukemia (AML) [11]. We recently revealed that *MT1G* and *MT1A* are direct target genes of PU.1, and that their expressions are negatively regulated by PU.1 [12]. Thus far, no studies analyzing MT functions in myeloid cells have been published. As MT1G is one of the major isoforms in the MT family [7], [8], we analyzed the function of MT1G in myelopoiesis in the present study. As a result, we found that overexpression of *MT1G* inhibited the ATRA-induced myeloid differentiation of NB4 cells.

## Materials and Methods

### Plasmids

To generate an MT1G expression vector, pcDNA-*MT1G* was constructed using the following primers, 5′-CTAGGAATTCTCGCCTCGGGTGCAATG-3′ and 5′-GCCCAAGCTTGGCGCAGCAGCTGCACTTCT-3′. The amplified DNA fragment was digested with EcoRI and HindIII, and inserted into the EcoRI/HindIII site of pcDNA 3.1/myc-His(-) version A (Invitrogen, Carlsbad, CA).

### Cell culture and generation of MT1G-overexpressing cells

To generate MT1G-overexpressing cells and their control cells, the *MT1G* expression vector and its parental pcDNA 3.1/myc-His(-) version A vector (Invitrogen) were transfected using a CLB-Transfection device (Lonza, Basel, Switzerland). NB4 clones stably transfected with the vectors were isolated by limiting dilution and selection with 400 µg/ml of neomycin in RPMI (Gibco BRL, Rockville, MD) containing 10% heat-inactivated fetal bovine serum (HIFBS). Cells were cultured under 5% CO_2_ at 37°C in a humidified atmosphere.

### Microarray and mRNA expression analyses

For RNA preparation for real-time PCR analyses, MT1G-overexpressing (NB4MTOE) cells and their control cells were seeded at a density of 1×10^5^ cells/ml and treated with 1 µM all-trans retinoic acid (ATRA) or an equal volume of its solvent (ethanol). The cells were harvested after 72 h, or at specified times. For microarray analyses, total cellular RNA was isolated from control (NB4pcDNA4, 6, 7) cells and NB4MTOE (NB4MT22, 23, 25) cells using an RNA Mini Purification Kit (Qiagen, Miami, FL) according to the manufacturer's protocol. Aliquots containing 10 µg of RNA from each sample of control cells were mixed and used as controls. Similarly, 10 µg of RNA from each sample of NB4MTOE cells were mixed and used as NB4MTOE cells. The samples were subjected to microarray analyses using a CodeLink Human 54K Whole Genome Bioarray (Filgen, Nagoya, Japan). The gene expression datasets have been deposited in the NCBI Gene Expression Omnibus (http://www.ncbi.nlm.nih.gov/geo/) and are accessible through the GEO series accession number GSE56739. For mRNA expression analyses, cDNAs were prepared from the cells using a Transcriptor First Strand cDNA Synthesis Kit (Roche, Indianapolis, IN). Quantitative PCR was performed using the Quantitect SYBR Green PCR Reagent (Qiagen) according to the manufacturer's protocol and an Opticon Mini Real-time PCR Instrument (Bio-Rad, Hercules, CA) as previously described [13]. The sequences and conditions of the primers used for real-time quantitative PCR are listed in Table 1. The copy number of each sample was calculated as previously described [14].

**Table 1:** Sequences and conditions for the primers used for real-time quantitative PCR.

Gene	Sequences	Conditions
*MT1G*	5′-CTTCTCGCTTGGGAACTCTA-3′	(A)
	5′-AGGGGTCAAGATTGTAGCAAA-3′	
*PU.1*	5′-GTGCCCTATGACAACGGATCT-3′	(B)
	5′-GAAGCTCTCGAACTCGCTGT-3′	
*CD11c*	5′-ACCACAAGCAGTAGCTCCTTC-3′	(A)
	5′-AAGTAGGAGCCGATCTGAGTC-3′	
*p21^WAF1/CIP1^*	5′-AGTGGACAGCGAGCAGCTGA-3′	(A)
	5′-TAGAAATCTGTCATGCTGGTCTG-3′	
*CD11b*	5′-GCCGGTGAAATATGCTGTCT-3′	(A)
	5′-GCGGTCCCATATGACAGTCT-3′	
*MPO*	5′-TCCTTCGTCACTGGCGTCA-3′	(A)
	5′-ATGCAGTCGGCTTGGTTCTT-3′	
*c-myc*	5′-AAGACTCCAGCGCCTTCTCTCCGT-3′	(A)
	5′-TGGGCTGTGAGGAGGTTTGCTGTG-3′	
*MMP-8*	5′-CCCAATGGAATCCTTGCTCA-3′	(A)
	5′-ATCAAGGCACCAGGGTCAGA-3′	
*S100A12*	5′-CTCCACATTCCTGTGCATTG-3′	(A)
	5′-TGCAAGCTCCTTTGTAAGCA-3′	
*OSM*	5′-AGTACCGCGTGCTCCTTG-3′	(A)
	5′-CCCTGCAGTGCTCTCTCAGT-3′	
*NCF 2*	5′-AATCGACAAGGCGATGGAGT-3′	(A)
	5′-GGGCAAACCCAGAGAAACTG-3′	
*azurocidin 1*	5′-CCCCTTTTGGACATCGTTGG-3′	(A)
	5′-CAGGTCATAGGCACCCAGCA-3′	
*defensin α4*	5′-GCCCTGCCTAGCTTGAGGAT-3′	(A)
	5′-TCTGCAAGAGCAGACCATGC-3′	
*PYCR 1*	5′-GACCTGGCCACAGTTTCTGCT-3′	(A)
	5′-CTCAATGTCGGCGCCTATTTC-3′	
*CXCR 3*	5′-CTGGCAGATCCAGAGGTTCC-3′	(A)
	5′-ACAGGCCTCAGCCAAATCAT-3′	
*GATA 2*	5′-ATCAAGCCCAAGCGAAGACT-3′	(A)
	5′-CATGGTCAGTGGCCTGTTAAC-3′	
*GAPDH*	5′-GAAGGTGAAGGTCGGAGT-3′	(A)
	5′-GAAGATGGTGATGGGATTTC-3′	

(A) 95°C for 15 min, followed by 35 cycles of 95°C for 30 s, 55°C for 30 s, and 72°C for 30 s.

(B) 95°C for 15 min, followed by 35 cycles of 95°C for 15 s and 60°C for 1 min.

### Differentiation assay

In each experiment, NB4 transgenic cells in the logarithmic growth phase were seeded at 2×10^5^ cells/ml, and induced to differentiate by 1 µM ATRA (Sigma, St. Louis, MO). The differentiated cells were collected for analysis at specified times. The differentiation was evaluated by the morphology after Wright–Giemsa staining, the nitroblue tetrazolium (NBT) reduction assays, and marker expression analyses by real-time PCR and flow cytometry. For the NBT reduction test, 5×10^5^ cells were incubated in 0.5 ml of a freshly prepared solution containing PBS, NBT (Sigma; 1 tablet/10 ml of PBS), and 0.33 µM PMA for 30 min at 37°C. After blind labeling of each sample, at least 200 cells were counted and the percentage of NBT-positive cells was calculated. Images were taken using an Olympus BX41 microscope and a DP70 digital camera with a DP controller system (Olympus Co., Tokyo, Japan).

### Cell cycle analysis

Cell cycle profiles were determined by analyzing the DNA contents using propidium iodide (Dojindo, Kumamoto, Japan) staining and flow cytometry. To assess the differentiation, cells were incubated with 1 µM ATRA for various times. Then, the cells were washed with PBS, fixed with ice-cold 70% ethanol, and stored at −20°C until analysis. The fixed cells were collected, suspended in 300 µl of RNase/PBS solution (100 µg/ml) and incubated at 37°C for 30 min. The cells were collected, resuspended in 300 µl of propidium iodide/PBS solution (5 µg/ml), and incubated in the dark at room temperature for 15 min. After filtration through a nylon mesh sheet, all samples were applied to an Epics XL (Beckman Coulter, Nyon, Switzerland). To analyze the data, WinMDI2.9 and MultiCycle AV for Windows (Phoenix, San Diego, CA) were employed as described [15].

### Surface marker expression analysis by flow cytometry

For flow cytometry analysis, the cells were washed twice with PBS, and 100-µl aliquots of the cell suspensions were protected from light and incubated with 10 µl of a PE-conjugated mouse anti-human CD11b antibody (BioLegend, San Diego, CA) for 60 min at room temperature. Isotype-matched PE-conjugated mouse IgG (BioLegend) antibody was used as a negative control. After the incubation, the samples were applied to a MACSQuant Analyzer (Miltenyi Biotec, Bergisch Gladbach, Germany). The mean fluorescence intensity (MFI) was calculated by subtracting the value of the mean fluorescence channel of the respective isotype control from the value obtained from the sample incubated with the specific antibody.

### Western blotting

To detect MT1G expression, nuclear extracts were employed as follows. Approximately 1×10^7^ cells were lysed in buffer A (10 mM Hepes, 10 mM KCl, 1.5 mM MgCl_2_, 1× phosphatase inhibitor cocktail (Roche), 1× protease inhibitor cocktail (Roche)) for 10 min on ice. After centrifugation at 1300×*g* for 10 min, the pellets were washed with buffer B (20 mM Hepes, 420 mM NaCl, 25% glycerol, 1.5 mM MgCl_2_, 0.2 mM EDTA, 0.5 mM DTT, 1× phosphatase inhibitor cocktail, 1× protease inhibitor cocktail) and resuspended. The lysates were subjected to ultrasonic sonication, followed by centrifugation at 8000×*g* for 15 min and collection of the supernatants. Aliquots of the supernatants containing 20–30 µg of protein were separated in a Tris-tricine gel (Bio-Rad), transferred to Sequi-blot PVDF membranes (Bio-Rad), and immunoblotted. To detect cell cycle-related proteins, total cellular extracts were prepared and immunoblotted as described [16]. To examine the expression of exogenous MT1G, a rabbit polyclonal metallothionein antibody (FL-61) (Santa Cruz, Santa Cruz, CA) was used. To examine the expressions of p21, cyclin D1, and cyclin A, specific rabbit polyclonal antibodies were used (Cell Signaling Technology, Beverly, MA). To verify equal loading of proteins in each lane, anti-histone H3 (for nuclear extracts) and anti-GAPDH (for total cellular extracts) rabbit polyclonal antibodies (Cell Signaling Technology) were also employed.

## Results

### Generation of MT1G overexpressing NB4 cells

**Figure 1:**
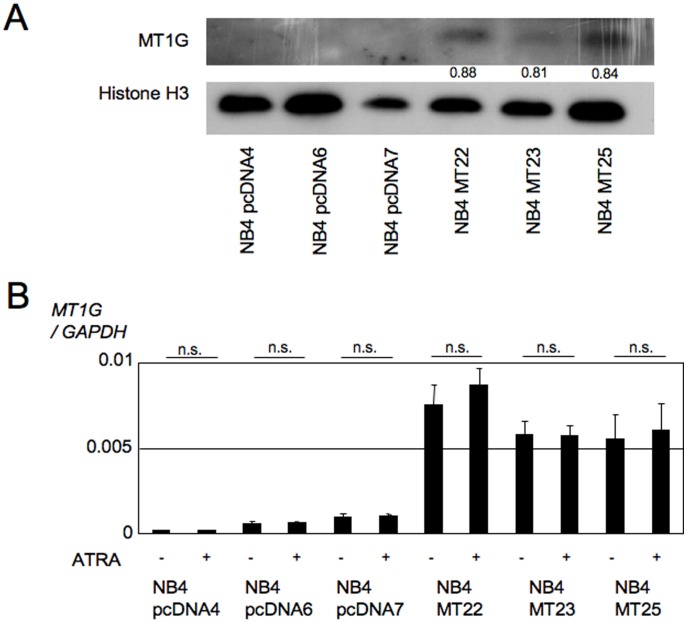
Establishment of NB4MTOE cells. (A) Expression of MT1G in NB4MTOE cells examined by western blotting. A rabbit polyclonal anti-MT antibody was used to detect exogenous MT1G. Equal amounts of soluble proteins were loaded in each lane and immunoblotted for MT and histone H3. The indicated numbers show the relative density, calculated with Image J 1.46 software, obtained as the density of each MT1G band divided by that of the corresponding histone H3 band. (B) The expression of *MT1G* was examined by real-time PCR (mean±SD; n.s., not significant). NB4MTOE cells and their control cells were cultured with or without 1 µM ATRA for 72 h, and then collected for analysis. Each gene transcript level was adjusted by the corresponding expression of *GAPDH*, and the relative levels are shown. The data presented were obtained from three independent PCR amplifications, and the reproducibility was confirmed by independent real-time PCR amplifications using different batches of cDNA.

To clarify whether MT1G functionally contributes to myeloid cell differentiation, an *MT1G* cDNA was cloned into pcDNA3.1, and the vector was electroporated into NB4 cells. The resulting clones were isolated by limiting dilution with medium containing neomycin. Among the >20 lines obtained, three clones exhibited sufficient expression levels of *MT1G* (Fig. 1A, B). Thereafter, clones NB4MT22 and NB4MT23 were mainly used to assess the effects of *MT1G* overexpression, and were designated NB4 MT1G-overexpressing (NB4MTOE) cells. In addition, we checked the expression of *MT1G* in NB4MTOE cells and their control cells during ATRA-induced NB4 cell differentiation. As a result, we found that there were no obvious changes following the addition of ATRA in either control or NB4MTOE cells (Fig. 1B).

### MT1G overexpression disturbs ATRA-induced expression changes of myeloid differentiation markers

**Figure 2:**
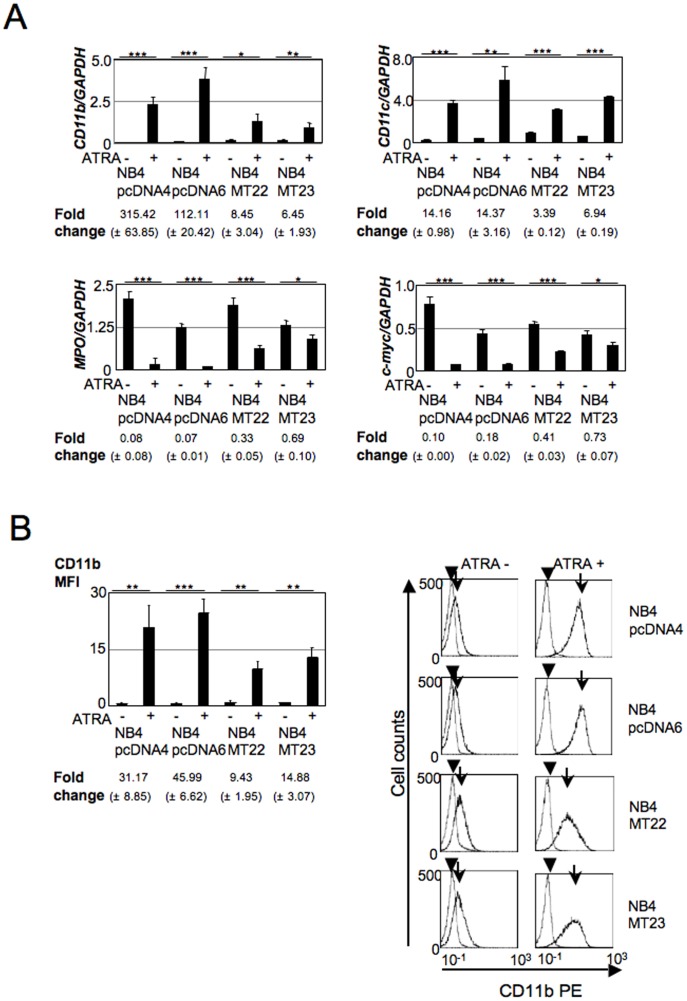
Analyses of ATRA-induced differentiation of NB4MTOE cells by differentiation markers. (A) The ATRA-induced differentiation of NB4MTOE cells and their control cells was examined by real-time PCR analysis of several markers. The expressions of *CD11b* (upper left), *CD11c* (upper right), *MPO* (lower left), and *c-myc* (lower right) were examined. The data shown were obtained from three independent PCR amplifications (mean±SD; *p<0.05; **p<0.01; ***p<0.001). In addition, the fold changes (mean±SD) are shown at the bottom of each panel. The fold changes were calculated as the relative expression of each marker in the ATRA-treated cells divided by the mean expression of the same marker in the ATRA-untreated cells. The p-values for the significance of the differences in the fold changes, calculated by Student's *t*-test, between control cells and NB4MTOE cells were: *CD11b*, p = 0.0017; *CD11c*, p<0.001; *MPO*, p<0.001; *c-myc*, p<0.001. (B) Effects of ATRA on the expression of CD11b in NB4 cells analyzed by flow cytometry. Shown on the left are the average ± standard deviations of the MFI for CD11b in the absence or presence of ATRA (1 µM) (*p<0.05; **p<0.01; ***p<0.001). Four independent experiments were performed and the data shown are calculated from these experiments. The fold changes (mean±SD) are shown at the bottom of the panel. The fold changes were calculated as the relative expressions of the MFI for CD11b in the ATRA-treated cells divided by the mean expression in the ATRA-untreated cells. The p-values for the significance of the differences in the fold changes, calculated by Student's *t*-test, between control cells and NB4MTOE cells were p<0.001. Right panels are representative histograms of these experiments. Arrows indicate the CD11b expression while arrowheads indicate control PE-IgG.

After establishing NB4MTOE cells, we examined whether the overexpression of MT1G could alter ATRA-induced NB4 cell differentiation. First, we examined several differentiation markers. Real-time PCR analyses demonstrated that the inductions of *CD11b* and *CD11c* and reductions in myeloperoxidase (*MPO*) and *c-myc* by ATRA were significantly attenuated in NB4MTOE cells (Fig. 2A). We further confirmed the findings for the reduced CD11b expression by flow cytometry analysis and confirmed the real-time PCR result for CD11b (Fig. 2B).

### MT1G overexpression impairs ATRA-induced G1 arrest in NB4 cells

**Figure 3:**
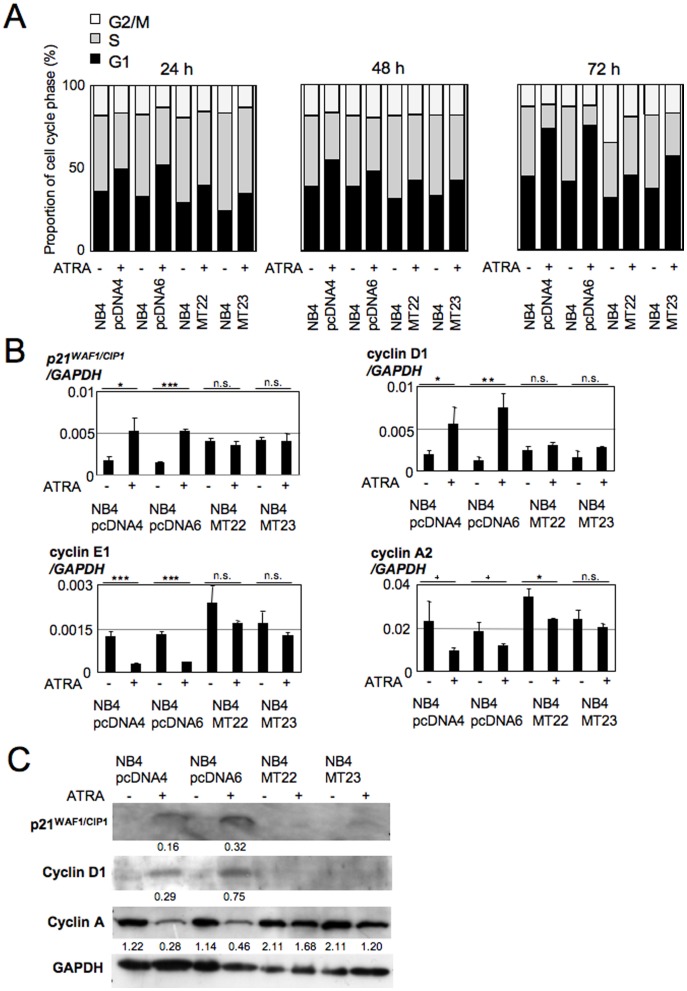
Changes in the cell cycle induced by ATRA in NB4MTOE cells. (A) Cell cycle profiles of NB4MTOE cells and their control cells in the presence (1 µM) or absence (solvent, ethanol) of ATRA for 72 h. At least three independent experiments were performed with similar results, and summaries of the percentages of cells in each phase of the cell cycle (G1, S, and G2/M) are shown. (B) Cell cycle marker expression changes induced by ATRA in NB4MTOE cells and their control cells. The expressions of *p21^WAF1/CIP1^* (upper left), *cyclin D1* (upper right), *cyclin E1* (lower left), and *cyclin A2* (lower right) were examined by real-time PCR (mean±SD; n.s., not significant; **^+^**p<0.1; *p<0.05; **p<0.01; ***p<0.001). The data presented were obtained from three independent PCR amplifications, and the reproducibility was confirmed by independent real-time PCR from different batches of cDNA. (C) Cell cycle marker expression changes induced by ATRA in NB4MTOE cells examined by western blotting. The indicated numbers are the relative density obtained as the density of the marker band (p21^WAF1/CIP1^, cyclin D1, cyclin A) divided by the density of the corresponding GAPDH band.

As G1 arrest is a hallmark of ATRA-induced NB4 cell differentiation [17], we next examined the cell cycle distributions of NB4MTOE cells and their control cells with or without ATRA. As a result, we observed decreases in G1 accumulation by ATRA in NB4MTOE cells throughout the time course (24–72 h) (Fig. 3A). Consistent with this, the inductions of the G1 regulator *p21^WAF1/CIP1^* and *cyclin D1* by ATRA were significantly attenuated in NB4MTOE cells (Fig. 3B, C). In addition, the reductions in cyclin E1 and cyclin A2 were also significantly attenuated in these cells (Fig. 3B, C). These findings suggest that MT1G impairs ATRA-induced G1 arrest.

### MT1G overexpression reduces the proportion of NBT positive cells

**Figure 4:**
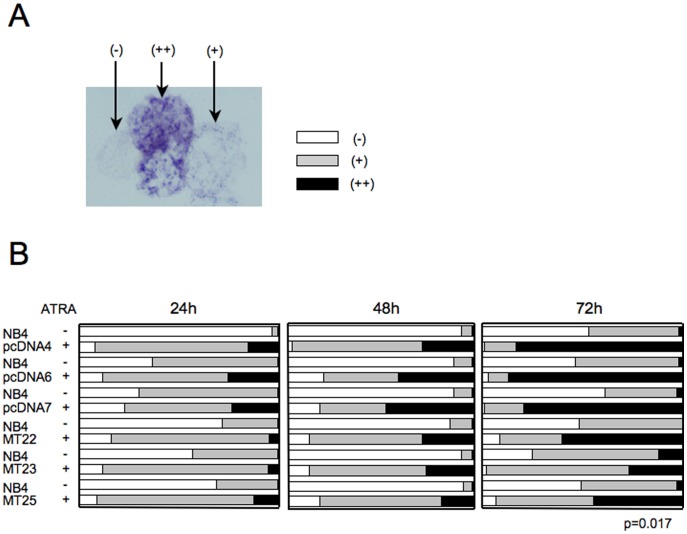
Analyses of ATRA-induced differentiation of NB4MTOE cells by NBT assay. (A) Assessment of NBT assay with or without ATRA. Examples of strongly positive (++), weakly positive (+), and negative (-) cells in the NBT reduction test. (B) Assessment of NBT assay in NB4MTOE cells and control cells. Cells were treated with 1 µM ATRA for 24 h (left), 48 h (middle), or 72 h (right panel). Black bars: NBT strongly positive cells (++); gray bars: NBT weakly positive cells (+); white bars: NBT negative (-) cells. After blind labeling of each sample, at least 200 cells were counted and the percentage of NBT-positive cells was calculated. The indicated p-value was calculated for the difference between the percentages in the (++) control (NB4pcDNA4, 6, 7) cells and NB4MTOE (NB4MT22, 23, 25) cells treated with ATRA for 72 h.

We further evaluated the role of MT1G in NB4 cell differentiation by performing NBT reduction assays. This assay is based on the ability of phagocytic cells to produce superoxide upon stimulation with phorbol 12-myristate 13-acetate (PMA), which is comparable to that generated by normal peripheral blood granulocytes [18]. Increased amounts of NBT-positive cells were observed in ATRA-induced control cells, even at 24 h after the addition of the reagent (Fig. 4A, B). Notably, at 72 h, there were significant reductions in NBT-positive cells in NB4MTOE cells compared with the control cells (Fig. 4A, B).

### Percentages of differentiated cells induced by ATRA are reduced in NB4MTOE cells

**Figure 5:**
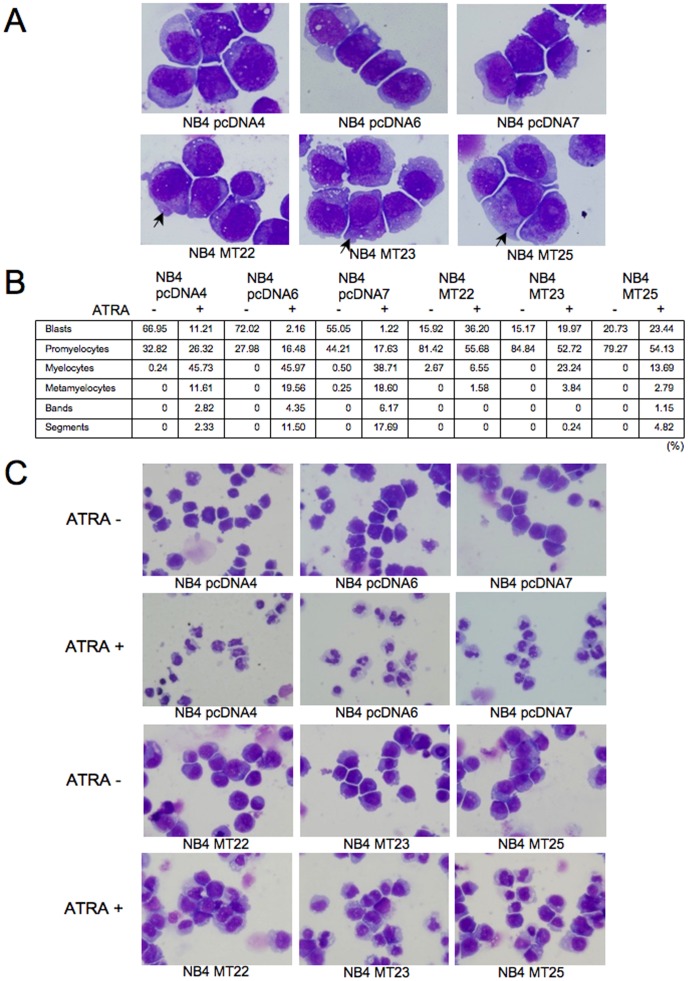
Morphologic analyses of NB4MTOE cells and their control cells. (A) NB4MTOE (NB4MT22, 23, 25) cells and their control (NB4pcDNA4, 6, 7) cells were collected and subjected to Wright–Giemsa staining. The magnification of the images shown in the panels is ×1000. Arrows indicate cytoplasmic granules. (B) Differential counts of NB4MTOE cells and their control cells in the presence or absence of ATRA. Cells were treated with 1 µM ATRA or solvent (control) for 5 days. After collection, the cells were subjected to Wright–Giemsa staining, and classified. This experiment was performed under blind labeling of each sample, with two licensed examiners. The two examiners counted more than 200 cells for each sample and obtained similar results. The percentages of the cells are the mean values of the data from the two examiners. (C) Aliquots of cells with or without ATRA treatment shown in panel B were examined for morphological changes by Wright–Giemsa staining. The magnification of the images shown in the panels is ×400.

Next, we examined the morphology of the cells with or without ATRA. First, we found that NB4MTOE (NB4MT22, 23, 25) cells were rich in cytoplasmic granules (Fig. 5A), with increased percentages of promyelocytic like cells (Fig. 5A, B) compared with their control cells in the absence of ATRA. We then compared the morphology of these cells after ATRA-induced differentiation. As a result, ATRA induced a significant increase in differentiated cells among control (NB4pcDNA4, 6, 7) cells, with indentation and bending of the nuclei and decreases in the cellular size and nuclear/cytoplasmic ratio, characteristic of metamyelocytes and the band stage of granulocytic differentiation (Fig. 5B, C). In sharp contrast, NB4MTOE cells showed reduced percentages of these differentiated cells (Figure 5B, C). These findings suggest that the overexpression of MT causes a change in the morphology accompanied by increased cytoplasmic granules, and reduced percentages of differentiated cells with ATRA.

### Gene profiling between NB4MTOE cells and their control cells in the presence of ATRA

**Figure 6:**
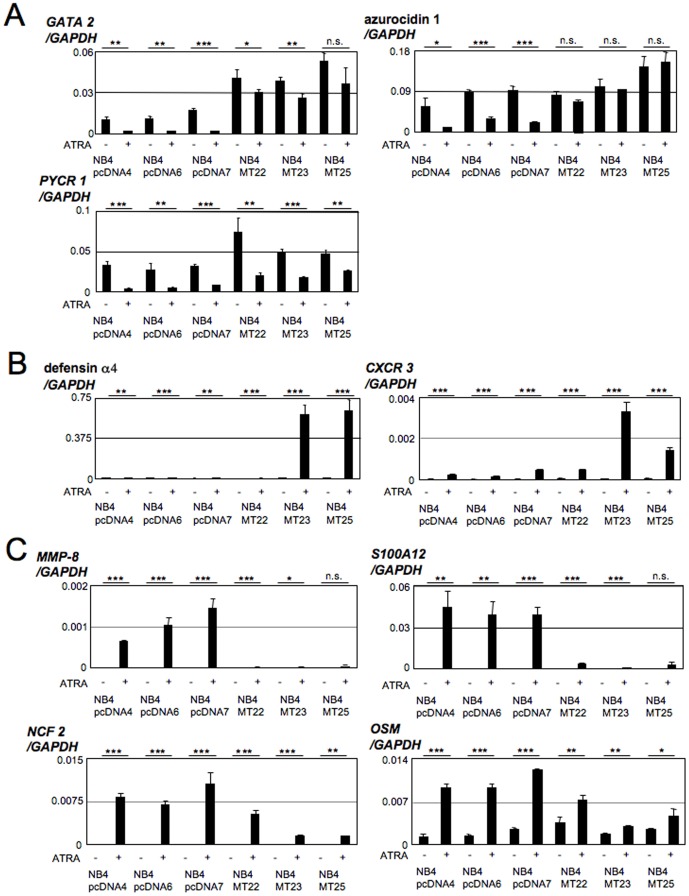
Validation of the microarray data on selected genes. Total RNAs from control and NB4MTOE cells cultured in the presence or absence of ATRA for 72-time PCR with specific primers. (A) Genes downregulated by ATRA with insufficient suppression in NB4MTOE cells. (B) Genes upregulated by ATRA with aberrant induction in NB4MTOE cells. (C) Genes upregulated by ATRA with insufficient induction in NB4MTOE cells. The data presented were obtained from three independent PCR amplifications and the reproducibility was confirmed using different batches of cDNA (mean±SD; n.s., not significant; *p<0.05; **p<0.01; ***p<0.001).

To investigate the effects of MT overexpression on ATRA-induced NB4 cell differentiation, we compared the transcriptomes between ATRA-treated NB4MTOE (NB4MT22, 23, 25) cells and their control (NB4pcDNA4, 6, 7) cells. RNA derived from both cell types was used for expression profiling with a CodeLink Human Whole Genome Bioarray. A total of 130 genes demonstrated significant alterations (greater than three-fold change) in expression between ATRA-induced NB4MTOE cells and the control cells. Among these, 52 genes were upregulated (Table S1) and 78 genes were downregulated (Table S2) by ATRA in NB4MTOE cells. In addition to the genes already confirmed in the differentiation assays (*MPO* and *p21^WAF1/CIP1^*), we investigated several other genes in the list that are known to have functions in myeloid cells: *GATA 2*; *azurocidin 1*; *pyrroline-5-carboxylate reductase* (*PYCR) 1*; *defensin α4*; *chemokine (C-X3-C motif) receptor (CXCR) 3*; *matrix metallopeptidase* (*MMP)-8*; *S100A12*; *neutrophil cytosolic factor* (*NCF*) *2*; and *oncostatin M* (*OSM*). The expression changes for all of these genes were verified using real-time PCR. GATA 2 is a hematopoietic transcription factor that is strictly expressed in immature hematopoietic cells [19]. We found that *GATA 2* expression was reduced in ATRA-treated control cells, but this reduction was tended to be disturbed in NB4MTOE cells (Fig. 6A). Azurocidin 1 is a component of azurophil granule protein, and the expression of this gene disappears in CD34^+^CD38^+^ cells after 5 days of culture in the presence of G-CSF [20]. Consistent with this, the expression of this gene was decreased by ATRA in control cells, while the decrease was smaller, or rather increased, in NB4MTOE cells (Fig. 6A). PYCR1 is a proline metabolic enzyme that modulates the NAD(P)H/NAD(P) ratio in the cytoplasm and mitochondria [21]. We observed that *PYCR1* expression was reduced by ATRA, but the reduction was slightly attenuated in NB4MTOE cells (Fig. 6A). For some myeloid genes, however, enhanced expression was observed in some of the NB4MTOE cells. Defensin α4 is one of the defensin family antimicrobial peptides [22]. CXCR3 is a G-protein-coupled 7-transmembrane receptor, and its expression is highly upregulated by GM-CSF on CD34^+^ human cord blood cells during myeloid cell differentiation [23]. We found that the inductions of *defensin α4* and *CXCR3* by ATRA were rather enhanced in NB4MTOE cells, NB4MT23 and NB4MT25 cells (Fig. 6B). However, this induction was not observed in NB4MT22 cells, possibly indicating that the potent inductions of these genes are not common features of MT-overexpressing cells. Furthermore, the inductions of several myeloid genes (*MMP-8*, *S100A2*, *NCF 2*, and *OSM*) by ATRA were profoundly impaired (Fig. 6C). MMP-8, also known as collagenase-2 or neutrophil collagenase, is mainly produced by neutrophils, and loss of this protease causes important deficiencies in the inflammatory responses induced by carcinogens [24]. S100A12 is a member of the S100 family of EF-hand calcium-binding proteins. S100A12 is predominantly expressed and secreted by neutrophil granulocytes, and induced by inflammation [25]. NCF 2 is a component of the leukocyte NADPH oxidase complex that produces superoxide [26]. OSM induces differentiation in myeloid cells and is mainly produced by activated T lymphocytes, monocytes, and macrophages [27]. We found that these genes were potently induced by ATRA in control cells, and that these inductions were abrogated in NB4MTOE cells (Fig. 6C). Collectively, overexpression of MT1G inhibits the normal differentiation-inducing effects of ATRA in NB4 cells.

## Discussion

In the present study, we observed that the ATRA-induced myeloid differentiation of NB4 cells was inhibited by overexpression of MT1G. p53 plays a role in myeloid differentiation through the upregulation of senescence-related genes, such as p21^WAF1/CIP1^ [28] or hypermethylated in cancer 1 transcription factor, which are involved in myeloid differentiation [29]. We speculate that the mechanisms of the impairment of the ATRA-induced differentiation in NB4MTOE cells might be dependent, at least in part, on the inhibition of p53 and/or related gene functions. By cotransfection experiments using a p53-dependent reporter gene with *p53* and *MT* expression vectors, Meplan *et al*. [30] demonstrated that MT exerts a potent inhibitory effect on p53 transcriptional activity when transfected with an excess of *MT* over *p53*, consistent with the metal chelator effect of MT. In a previous study, analyses of *p53* ^-/-^ and *GATA2* ^-/-^ compound-mutant embryos showed that the absence of *p53* was able to partially restore the total number of *GATA2* ^-/-^ hematopoietic cells [31]. This finding suggests that inhibition of p53 can rescue the function of *GATA2*. Consistently, we observed that overexpression of MT1G resulted in increased basal *GATA2* expression (Fig. 6A), which may also indicate the involvement of p53 inhibition through MT1G. Kondo *et al*. [32] demonstrated that mouse embryonic cells null for *MT1* and *MT2* were more susceptible to apoptotic death after exposure to anticancer agents cytosine arabinoside, bleomycin, melphalan, or cis-dichlorodiammineplatinum (II) compared with wild-type cells. The p53 protein levels were highest in *MT* null cells, which may also suggest a role for an inhibitory function of MT toward p53. Another possible mechanism for the inhibition of myeloid differentiation by MT may be involvement of ROS regulation. Since MT possesses potent antioxidant functions [2] and the generation of reactive oxygen species is important for the function of neutrophils for antibacterial activity, aberrant ROS regulation by the overexpression of MT might be playing a role in the differentiation block in these cells. We are now analyzing these mechanisms for future clarification.

We observed that MT1G-overexpressing cells had an increased percentage of promyelocytic cells rich in cytoplasmic granules. It was previously reported that addition of zinc to human neutrophils inhibited azurophil granule secretion in response to several stimulants such as monensin and zymosan [33]. This is consistent with our finding that the overexpression of MT, a potent zinc chelator, induced cytoplasmic azurophil granules. Accordingly, we revealed that azurocidin 1, a component of azurophil granules, and its disappearance were necessary for granulocytic differentiation, and that its reduction by ATRA was impaired in NB4MTOE cells (Fig. 6). Therefore, it is possible to speculate that the deregulation of azurophil granule expression by MT also plays a role in the inhibition of myeloid differentiation.

Few studies have analyzed the roles of MT in hematopoiesis [1]. In the erythroid lineage, it has been reported EPO- or sodium butyrate-induced differentiation was inhibited in K562 cells stably transfected with an expression vector containing the human *MT2A* gene [34]. In the megakaryocytic lineage, it was recently reported that overexpression of *MT2A* in megakaryocytic DAMI cells caused increases in the cell size, intracellular granulation and levels of megakaryocytic-specific CD41 and CD42 with arrest of cell proliferation, suggesting a positive role for MT in megakaryocytic differentiation [35]. Considering the positive role of MT for differentiation in the megakaryocytic lineage [35] and the negative role of MT for erythroid differentiation [34], the roles of MT toward differentiation may differ in different lineages.

Mice lacking *MT1* and *2* have been generated in previous studies [36], [37]. Although some differences in phenotypes and metabolic responses may exist, susceptibility to heavy metal toxicity, responses to inflammation, and altered zinc homeostasis are common features of the phenotypes of these double-knockout mice. Despite the absence of these *MT* genes, it was reported that all of the blood lineages were still present [38]. However, Sugiura *et al*. [39] demonstrated dysfunction of the macrophages in *MT1/2* double-knockout mice. When compared with wild-type mice, the macrophages from *MT1/2* double-knockout mice showed defects in their phagocytic and antigen-presenting activity. In addition, the productions of cytokines such as IL-1α, IL-6, IL-10, and IL-12 were reduced in the macrophages from *MT*-knockout mice. Collectively, these findings suggest that complete loss of the expression of *MT* may also disturb the function of hematopoietic cells, at least in a certain lineage.

In AML, the expression of the resistance-related proteins P-glycoprotein 170 (P-170), glutathione-S-transferase pi (GST-Pi), topoisomerase-II (Topo II), thymidylate synthase (TS) and MT was investigated in leukemic cells from 19 children with newly diagnosed AML [40]. MT was expressed in leukemic cells from 68% of cases with newly diagnosed AML. Although the number of patients was small, they concluded that patients who developed relapse showed a poorer prognosis, and frequently expressed more than two resistance-related proteins, including MT, compared with patients who remained in remission [40]. As we revealed inverse correlations between *MT1G* and *PU.1* expression in AML patients [12], and we also previously reported that *PU.1* expression was inversely correlated with the tyrosine kinase receptor *FLT3* [13], and that strong expression of wild-type *FLT3* was an unfavorable prognostic factor for overall survival [41], [42]. In addition, PU.1 expression was reported to be a positive indicator for other hematological malignancies, such as follicular lymphoma [43]. Although further extensive analyses are required, it is possible that increased *MT1G* expression represents a poor prognostic marker for AML. These important issues need to be addressed in future investigations. Such future analyses may lead to the development of useful prognostic markers for myeloid malignancies.

## Supporting Information

Table S1 Candidate genes upregulated by ATRA in NB4MTOE cells compared with NB4pcDNA cells.
(DOC)

Table S2 Candidate genes downregulated by ATRA in NB4MTOE cells compared with NB4pcDNA cells.
(DOC)
