# Supplementary material for: A PU.1 Suppressive Target Gene, Metallothionein 1G, Inhibits Retinoic Acid-Induced NB4 Cell Differentiation
Source: PLoS One. 2014 Jul 29;9(7):e103282. doi: 10.1371/journal.pone.0103282 (PMC4114787; doi:10.1371/journal.pone.0103282)
Supplement: Table S1 — Candidate genes upregulated by ATRA in NB4MTOE cells compared with NB4pcDNA cells. (DOC) [file pone.0103282.s001.doc]

**Table S1:** Candidate genes upregulated by ATRA in NB4MTOE cells compared with NB4pcDNA cells

|  | GenBank Acc. No. | Gene name | Net intensity (MTOE) | Net intensity (pcDNA) | Net intensity (ratio) |
| --- | --- | --- | --- | --- | --- |
| 1 | AI207479 | chromosome 11 open reading frame 10 | 1625.7415 | 39.3145 | 41.352211 |
| 2 | NM_053044 | HtrA serine peptidase 3 | 264.81745 | 22.26475 | 11.894023 |
| 3 | NM_001276 | chitinase 3-like 1 (cartilage glycoprotein-39) | 399.4078 | 35.88435 | 11.130418 |
| 4 | BX448191 | - | 321.8858 | 32.7958 | 9.814848 |
| 5 | NM_032638 | **GATA binding protein 2** | 341.50255 | 37.3325 | 9.147594 |
| 6 | NM_001012631, NM_001012718, AI983408, NM_001012632, NM_001012635, NM_001012634, NM_004221, NM_001012636 | interleukin 32 | 711.4954 | 87.8662 | 8.097487 |
| 7 | NM_000237 | lipoprotein lipase | 225.30705 | 28.51045 | 7.902613 |
| 8 | AI689391, NM_001276 | chitinase 3-like 1 (cartilage glycoprotein-39) | 1475.18085 | 191.7939 | 7.69149 |
| 9 | NM_001673, NM_183356, NM_133436 | asparagine synthetase (glutamine-hydrolyzing) | 795.58225 | 127.155 | 6.256791 |
| 10 | NM_004926 | zinc finger protein 36, C3H type-like 1 | 127.4557 | 22.67115 | 5.621934 |
| 11 | NM_001700 | **azurocidin 1** | 601.3492 | 107.9929 | 5.568414 |
| 12 | CA310903, NM_001017928, BE568192 | coiled-coil domain containing 58 | 311.2826 | 57.571 | 5.406934 |
| 13 | NM_000250 | **myeloperoxidase** | 4284.20675 | 837.44045 | 5.115835 |
| 14 | NM_000523 | homeobox D13 | 137.5484 | 27.043 | 5.086285 |
| 15 | NM_031942, NM_145810 | cell division cycle associated 7 | 175.614 | 36.576 | 4.801345 |
| 16 | NM_178836 | - | 352.7231 | 74.35725 | 4.743628 |
| 17 | NM_058179, NM_021154 | phosphoserine aminotransferase 1 | 555.88275 | 120.4915 | 4.61346 |
| 18 | NM_001091 | amiloride binding protein 1 (amine oxidase (copper-containing)) | 295.1186 | 67.7019 | 4.359089 |
| 19 | NM_006623, BQ877146 | - | 272.7045 | 65.6826 | 4.151853 |
| 20 | NM_002777 | proteinase 3 | 1664.00565 | 405.91415 | 4.099403 |
| 21 | NM_001200 | bone morphogenetic protein 2 | 174.8177 | 44.6151 | 3.918353 |
| 22 | NM_130469 | Jun dimerization protein 2 | 445.7434 | 115.46415 | 3.860448 |
| 23 | NM_019042 | pseudouridylate synthase 7 homolog (S. cerevisiae) | 187.98975 | 48.78315 | 3.85358 |
| 24 | NM_152388 | transmembrane protein 237 | 207.8898 | 54.8596 | 3.789488 |
| 25 | NM_016938 | EGF containing fibulin-like extracellular matrix protein 2 | 120.6076 | 32.25735 | 3.738918 |
| 26 | NM_001925 | **defensin, alpha 4, corticostatin** | 1571.31845 | 422.41005 | 3.719889 |
| 27 | BI520935, NM_000349, NM_001007243 | - | 162.9076 | 43.8995 | 3.710922 |
| 28 | BQ576279, NM_006907, NM_153824 | **pyrroline-5-carboxylate reductase 1** | 147.704 | 41.0266 | 3.600201 |
| 29 | NM_005833 | Rab9 effector protein with kelch motifs | 324.1211 | 90.08695 | 3.59787 |
| 30 | AK095367 | G1 to S phase transition 1 | 412.7544 | 116.7675 | 3.53484 |
| 31 | NM_001337 | **chemokine (C-X3-C motif) receptor 1** | 96.17015 | 27.214 | 3.533848 |
| 32 | NM_002101, NM_016815 | glycophorin C (Gerbich blood group) | 1445.06875 | 409.3085 | 3.530512 |
| 33 | NM_006993 | nucleophosmin/nucleoplasmin 3 | 185.9869 | 53.60655 | 3.469481 |
| 34 | BQ002637 | uncharacterized LOC100506776 | 235.9136 | 68.57025 | 3.440466 |
| 35 | NM_005567 | lectin, galactoside-binding, soluble, 3 binding protein | 799.51455 | 232.9147 | 3.43265 |
| 36 | NM_001565 | chemokine (C-X-C motif) ligand 10 | 1723.63615 | 503.32215 | 3.424519 |
| 37 | NM_001252 | CD70 molecule | 896.5179 | 263.15715 | 3.406778 |
| 38 | AI631285, NM_014608 | - | 466.987 | 137.61145 | 3.393518 |
| 39 | NM_003690 | protein kinase, interferon-inducible double stranded RNA dependent activator | 333.9306 | 101.4712 | 3.29089 |
| 40 | NM_006452 | phosphoribosylaminoimidazole carboxylase, phosphoribosylaminoimidazole succinocarboxamide synthetase | 1488.607 | 452.82475 | 3.28738 |
| 41 | NM_014059 | regulator of cell cycle | 70.00115 | 21.38325 | 3.273644 |
| 42 | NM_002562, NM_177427 | purinergic receptor P2X, ligand-gated ion channel, 7 | 85 | 26.327 | 3.228625 |
| 43 | NM_002528 | nth endonuclease III-like 1 (E. coli) | 183.70935 | 58.04165 | 3.16513 |
| 44 | NM_005052 | ras-related C3 botulinum toxin substrate 3 (rho family, small GTP binding protein Rac3) | 81.7313 | 26.22295 | 3.116785 |
| 45 | BF984536 | talin 2 | 100.8434 | 32.39355 | 3.11307 |
| 46 | N47340 | - | 139.69835 | 44.9749 | 3.10614 |
| 47 | NM_177995, NM_152422 | protein tyrosine phosphatase domain containing 1 | 90.44525 | 29.38395 | 3.078049 |
| 48 | BM716531 | B7 homolog 6 | 119.99245 | 39.25395 | 3.056825 |
| 49 | NM_003078, NM_001003801, NM_001003802 | SWI/SNF related, matrix associated, actin dependent regulator of chromatin, subfamily d, member 3 | 121.74005 | 39.9571 | 3.046769 |
| 50 | NM_014867 | kelch repeat and BTB (POZ) domain containing 11 | 248.12995 | 81.75195 | 3.035156 |
| 51 | NM_030810, NM_022085 | thioredoxin domain containing 5 (endoplasmic reticulum) | 411.01655 | 135.9021 | 3.024358 |
| 52 | NM_004332, AV705828 | - | 79.9983 | 26.4686 | 3.022385 |
| 53 | BG388820, NM_006452 | - | 1298.2034 | 432.91235 | 2.998767 |
| 54 | NM_138773 | solute carrier family 25, member 46 | 79.1871 | 26.51185 | 2.986857 |
| 55 | BI520935, NM_000349, NM_001007243 | - | 102.79015 | 34.5217 | 2.977552 |
| 56 | NM_004044 | 5-aminoimidazole-4-carboxamide ribonucleotide formyltransferase/IMP cyclohydrolase | 378.60295 | 128.24885 | 2.952096 |
| 57 | NM_006138 | membrane-spanning 4-domains, subfamily A, member 3 (hematopoietic cell-specific) | 135.0052 | 46.03935 | 2.932387 |
| 58 | NM_002037, NM_153048, NM_153047 | FYN oncogene related to SRC, FGR, YES | 686.6938 | 235.2635 | 2.918828 |
| 59 | BC047421 | coiled-coil domain containing 163, pseudogene | 105.59605 | 36.3882 | 2.901931 |
| 60 | AI873847 | - | 55.28465 | 19.1543 | 2.886279 |
| 61 | NM_021095, BM041356 | solute carrier family 5 (sodium-dependent vitamin transporter), member 6 | 285.8129 | 99.0802 | 2.884662 |
| 62 | AI349547, NM_198175, NM_000269 | non-metastatic cells 1, protein (NM23A) expressed in | 1226.82665 | 425.89535 | 2.880582 |
| 63 | NM_001001549, NM_001001550, NM_001001555, NM_005311 | growth factor receptor-bound protein 10 | 64.78295 | 22.54035 | 2.874088 |
| 64 | X56789 | patatin-like phospholipase domain containing 2 | 306.03025 | 106.8186 | 2.864953 |
| 65 | NM_005950, H53339 | Metallothionein 1G | 634.4231 | 222.65635 | 2.849338 |

Table 2. Genes downregulated by ATRA in NB4MTOE cells compared with NB4pcDNA cells

|  |  |  |  |  |  |
| --- | --- | --- | --- | --- | --- |
|  |  |  |  |  |  |
|  |  |  |  |  |  |
|  |  |  |  |  |  |
|  |  |  |  |  |  |
|  |  |  |  |  |  |
|  |  |  |  |  |  |
|  |  |  |  |  |  |
|  |  |  |  |  |  |
|  |  |  |  |  |  |
|  |  |  |  |  |  |
|  |  |  |  |  |  |
|  |  |  |  |  |  |
|  |  |  |  |  |  |
|  |  |  |  |  |  |
|  |  |  |  |  |  |
|  |  |  |  |  |  |
|  |  |  |  |  |  |
|  |  |  |  |  |  |
|  |  |  |  |  |  |
|  |  |  |  |  |  |
|  |  |  |  |  |  |
|  |  |  |  |  |  |
|  |  |  |  |  |  |
|  |  |  |  |  |  |
|  |  |  |  |  |  |
|  |  |  |  |  |  |
|  |  |  |  |  |  |
|  |  |  |  |  |  |
|  |  |  |  |  |  |
|  |  |  |  |  |  |
|  |  |  |  |  |  |
|  |  |  |  |  |  |
|  |  |  |  |  |  |
|  |  |  |  |  |  |
|  |  |  |  |  |  |
|  |  |  |  |  |  |
|  |  |  |  |  |  |
|  |  |  |  |  |  |
|  |  |  |  |  |  |
|  |  |  |  |  |  |
|  |  |  |  |  |  |
|  |  |  |  |  |  |
|  |  |  |  |  |  |
|  |  |  |  |  |  |
|  |  |  |  |  |  |
|  |  |  |  |  |  |
|  |  |  |  |  |  |
|  |  |  |  |  |  |
|  |  |  |  |  |  |
|  |  |  |  |  |  |
|  |  |  |  |  |  |
|  |  |  |  |  |  |
|  |  |  |  |  |  |
|  |  |  |  |  |  |
|  |  |  |  |  |  |
|  |  |  |  |  |  |
|  |  |  |  |  |  |
|  |  |  |  |  |  |
|  |  |  |  |  |  |
|  |  |  |  |  |  |
|  |  |  |  |  |  |
|  |  |  |  |  |  |
|  |  |  |  |  |  |
|  |  |  |  |  |  |
|  |  |  |  |  |  |
|  |  |  |  |  |  |
|  |  |  |  |  |  |
|  |  |  |  |  |  |
|  |  |  |  |  |  |
|  |  |  |  |  |  |
|  |  |  |  |  |  |
|  |  |  |  |  |  |
|  |  |  |  |  |  |
|  |  |  |  |  |  |
|  |  |  |  |  |  |
|  |  |  |  |  |  |
|  |  |  |  |  |  |
|  |  |  |  |  |  |
