# Supplementary material for: A PU.1 Suppressive Target Gene, Metallothionein 1G, Inhibits Retinoic Acid-Induced NB4 Cell Differentiation
Source: PLoS One. 2014 Jul 29;9(7):e103282. doi: 10.1371/journal.pone.0103282 (PMC4114787; doi:10.1371/journal.pone.0103282)
Supplement: Table S2 — Candidate genes downregulated by ATRA in NB4MTOE cells compared with NB4pcDNA cells. (DOC) [file pone.0103282.s002.doc]

Table 1. Genes upregulated by ATRA in NB4MTOE cells compared with NB4pcDNA cells

|  |  |  |  |  |  |
| --- | --- | --- | --- | --- | --- |
|  |  |  |  |  |  |
|  |  |  |  |  |  |
|  |  |  |  |  |  |
|  |  |  |  |  |  |
|  |  |  |  |  |  |
|  |  |  |  |  |  |
|  |  |  |  |  |  |
|  |  |  |  |  |  |
|  |  |  |  |  |  |
|  |  |  |  |  |  |
|  |  |  |  |  |  |
|  |  |  |  |  |  |
|  |  |  |  |  |  |
|  |  |  |  |  |  |
|  |  |  |  |  |  |
|  |  |  |  |  |  |
|  |  |  |  |  |  |
|  |  |  |  |  |  |
|  |  |  |  |  |  |
|  |  |  |  |  |  |
|  |  |  |  |  |  |
|  |  |  |  |  |  |
|  |  |  |  |  |  |
|  |  |  |  |  |  |
|  |  |  |  |  |  |
|  |  |  |  |  |  |
|  |  |  |  |  |  |
|  |  |  |  |  |  |
|  |  |  |  |  |  |
|  |  |  |  |  |  |
|  |  |  |  |  |  |
|  |  |  |  |  |  |
|  |  |  |  |  |  |
|  |  |  |  |  |  |
|  |  |  |  |  |  |
|  |  |  |  |  |  |
|  |  |  |  |  |  |
|  |  |  |  |  |  |
|  |  |  |  |  |  |
|  |  |  |  |  |  |
|  |  |  |  |  |  |
|  |  |  |  |  |  |
|  |  |  |  |  |  |
|  |  |  |  |  |  |
|  |  |  |  |  |  |
|  |  |  |  |  |  |
|  |  |  |  |  |  |
|  |  |  |  |  |  |
|  |  |  |  |  |  |
|  |  |  |  |  |  |
|  |  |  |  |  |  |
|  |  |  |  |  |  |
|  |  |  |  |  |  |
|  |  |  |  |  |  |
|  |  |  |  |  |  |
|  |  |  |  |  |  |
|  |  |  |  |  |  |
|  |  |  |  |  |  |
|  |  |  |  |  |  |
|  |  |  |  |  |  |
|  |  |  |  |  |  |
|  |  |  |  |  |  |
|  |  |  |  |  |  |
|  |  |  |  |  |  |
|  |  |  |  |  |  |

**Table S2:** Candidate genes downregulated by ATRA in NB4MTOE cells compared with NB4pcDNA cells

|  | GenBank Acc. No. | Gene name | Net intensity (MTOE) | Net intensity (pcDNA) | Net intensity (ratio) |
| --- | --- | --- | --- | --- | --- |
| 1 | NM_000483 | apolipoprotein C-II | 20.814 | 423.2405 | 0.049178 |
| 2 | BC039398 | protocadherin 9 | 171.3854 | 2713.4346 | 0.063162 |
| 3 | AW337833 | **matrix metallopeptidase 8 (neutrophil collagenase)** | 36.4082 | 377.07385 | 0.096555 |
| 4 | BU177980 | - | 21.93185 | 220.64095 | 0.099401 |
| 5 | NM_014358 | C-type lectin domain family 4, member E | 146.864 | 870.17965 | 0.168774 |
| 6 | BC040958 | napsin B aspartic peptidase pseudogene | 42.1581 | 245.6416 | 0.171624 |
| 7 | NM_001462, NM_001005738 | formyl peptide receptor 2 | 33.25165 | 191.172 | 0.173936 |
| 8 | NM_003739 | aldo-keto reductase family 1, member C3 (3-alpha hydroxysteroid dehydrogenase, type II) | 27.46795 | 147.97675 | 0.185623 |
| 9 | AV715015 | cannabinoid receptor 2 (macrophage) | 21.1716 | 112.92875 | 0.187478 |
| 10 | NM_001974 | egf-like module containing, mucin-like, hormone receptor-like 1 | 74.20515 | 390.383 | 0.190083 |
| 11 | AI272963 | - | 24.17345 | 118.6508 | 0.203736 |
| 12 | NM_018515 | - | 38.74745 | 182.2066 | 0.212657 |
| 13 | NM_024850 | butyrophilin-like 8 | 64.3259 | 300.82395 | 0.213832 |
| 14 | NM_002029 | formyl peptide receptor 1 | 75.36185 | 346.1519 | 0.217713 |
| 15 | R23839 | adrenergic, beta-1-, receptor | 29.2897 | 133.02245 | 0.220186 |
| 16 | NM_180991 | solute carrier organic anion transporter family, member 4C1 | 209.9351 | 940.2356 | 0.223279 |
| 17 | AA868123 | small G protein signaling modulator 3 | 55.57665 | 248.37285 | 0.223763 |
| 18 | NM_006072 | chemokine (C-C motif) ligand 26 | 20.648 | 91.62115 | 0.225363 |
| 19 | R43301 | - | 29.18455 | 127.44285 | 0.229001 |
| 20 | NM_002575 | serpin peptidase inhibitor, clade B (ovalbumin), member 2 | 527.08665 | 2291.2427 | 0.230044 |
| 21 | NM_002964, AW440817 | S100 calcium binding protein A8 | 2536.91665 | 10885.65915 | 0.233051 |
| 22 | NM_175080, NM_002561 | purinergic receptor P2X, ligand-gated ion channel, 5 | 40.8621 | 174.3641 | 0.234349 |
| 23 | BM990501 | melan-A | 53.93685 | 227.40585 | 0.237183 |
| 24 | NM_173061, NM_173060, NM_001750, NM_173062 | calpastatin | 118.97975 | 498.59445 | 0.23863 |
| 25 | NM_177551, NM_006018 | - | 92.338 | 384.8846 | 0.239911 |
| 26 | BM683309 | - | 39.803 | 164.8032 | 0.241518 |
| 27 | NM_006163 | nuclear factor (erythroid-derived 2), 45kDa | 38.38155 | 158.46835 | 0.242203 |
| 28 | AI097618, NM_078467, NM_000389 | **cyclin-dependent kinase inhibitor 1A (p21, Cip1)** | 176.13885 | 721.24695 | 0.244214 |
| 29 | NM_002228 | jun proto-oncogene | 20.59735 | 83.03425 | 0.248058 |
| 30 | NM_005621 | **S100 calcium binding protein A12** | 1404.35525 | 5618.28025 | 0.249962 |
| 31 | NM_001828 | Charcot-Leyden crystal protein | 3842.81815 | 15304.771 | 0.251086 |
| 32 | NM_000655 | - | 184.71685 | 699.3973 | 0.264109 |
| 33 | NM_005874 | leukocyte immunoglobulin-like receptor, subfamily B (with TM and ITIM domains), member 2 | 58.93405 | 221.2889 | 0.266322 |
| 34 | NM_009588, NM_002341 | lymphotoxin beta (TNF superfamily, member 3) | 81.8859 | 303.6416 | 0.269679 |
| 35 | NM_020530 | **oncostatin M** | 35.22555 | 130.37045 | 0.270196 |
| 36 | L06895 | MAX dimerization protein 1 | 51.8822 | 190.6459 | 0.272139 |
| 37 | NM_181985, NM_021250 | leukocyte immunoglobulin-like receptor, subfamily A (with TM domain), member 5 | 175.193 | 635.19295 | 0.275811 |
| 38 | NM_003282 | troponin I type 2 (skeletal, fast) | 71.7919 | 258.1316 | 0.278121 |
| 39 | BC047362 | pleckstrin homology-like domain, family A, member 1 | 234.8753 | 836.97375 | 0.280624 |
| 40 | NM_032369 | hydrogen voltage-gated channel 1 | 395.52485 | 1406.84085 | 0.281144 |
| 41 | M90391 | interleukin 16 | 363.5201 | 1292.88995 | 0.281169 |
| 42 | NM_080817 | G protein-coupled receptor 82 | 392.9135 | 1396.8942 | 0.281276 |
| 43 | H99504 | thrombospondin 1 | 42.0247 | 148.31235 | 0.283353 |
| 44 | NM_019885 | cytochrome P450, family 26, subfamily B, polypeptide 1 | 189.1387 | 663.3692 | 0.285118 |
| 45 | NM_139241 | FYVE, RhoGEF and PH domain containing 4 | 29.90135 | 101.51975 | 0.294537 |
| 46 | NM_001007544 | chromosome 1 open reading frame 186 | 211.79115 | 715.33335 | 0.296073 |
| 47 | - | - | 4660.35445 | 15634.61425 | 0.298079 |
| 48 | NM_002284 | keratin 86 | 132.81235 | 443.7039 | 0.299327 |
| 49 | AW953756, NM_000576 | - | 1431.74315 | 4709.4856 | 0.304013 |
| 50 | AI096713 | - | 21.98335 | 71.38735 | 0.307945 |
| 51 | NM_153449, NM_006931 | - | 296.75115 | 959.54985 | 0.309261 |
| 52 | NM_007350 | pleckstrin homology-like domain, family A, member 1 | 93.22835 | 300.26185 | 0.31049 |
| 53 | NM_004120 | guanylate binding protein 2, interferon-inducible | 414.555 | 1333.19155 | 0.310949 |
| 54 | CA430431 | family with sequence similarity 207, member A | 41.31705 | 131.93105 | 0.313172 |
| 55 | NM_001007593, NM_000543 | sphingomyelin phosphodiesterase 1, acid lysosomal | 136.9569 | 435.8479 | 0.314231 |
| 56 | NM_013352 | dermatan sulfate epimerase | 71.5732 | 227.6341 | 0.314422 |
| 57 | NM_139314, NM_016109 | angiopoietin-like 4 | 393.1651 | 1239.2238 | 0.317267 |
| 58 | H62202 | melanoma antigen family D, 2 | 17.1278 | 53.93365 | 0.317572 |
| 59 | NM_006697, NM_181873 | myotubularin related protein 11 | 96.92745 | 303.58975 | 0.319271 |
| 60 | BX648180 | protein tyrosine phosphatase, receptor type, E | 290.8518 | 910.0155 | 0.319612 |
| 61 | NM_023944 | cytochrome P450, family 4, subfamily F, polypeptide 12 | 44.01 | 137.5371 | 0.319986 |
| 62 | NM_003059 | solute carrier family 22 (organic cation/ergothioneine transporter), member 4 | 56.05495 | 174.2638 | 0.321667 |
| 63 | NM_001781 | CD69 molecule | 46.0665 | 143.0779 | 0.321968 |
| 64 | AA633709, NM_021643 | - | 118.28675 | 367.08055 | 0.322236 |
| 65 | AL573083, NM_006821 | - | 133.43155 | 412.88745 | 0.323167 |
| 66 | NM_080657 | radical S-adenosyl methionine domain containing 2 | 70.13615 | 216.92545 | 0.323319 |
| 67 | NM_006343 | c-mer proto-oncogene tyrosine kinase | 55.894 | 172.63865 | 0.323763 |
| 68 | NM_013386, NM_213651 | solute carrier family 25 (mitochondrial carrier; phosphate carrier), member 24 | 26.33655 | 81.10475 | 0.324723 |
| 69 | NM_003656 | calcium/calmodulin-dependent protein kinase I | 170.75835 | 523.4421 | 0.326222 |
| 70 | NM_199004, NM_004313 | arrestin, beta 2 | 263.69885 | 806.7357 | 0.326871 |
| 71 | NM_005024 | serpin peptidase inhibitor, clade B (ovalbumin), member 10 | 38.7912 | 118.45535 | 0.327475 |
| 72 | NM_024430 | proline-serine-threonine phosphatase interacting protein 2 | 349.932 | 1068.2299 | 0.327581 |
| 73 | NM_145898, NM_005064 | chemokine (C-C motif) ligand 23 | 76.8769 | 234.589 | 0.327709 |
| 74 | CB155904, NM_001002235, NM_000295, NM_001002236 | - | 381.2704 | 1163.38275 | 0.327726 |
| 75 | NM_000433 | **neutrophil cytosolic factor 2** | 426.49335 | 1298.2034 | 0.328526 |
| 76 | AA918686 | 6-phosphofructo-2-kinase/fructose-2,6-biphosphatase 2 | 24.1514 | 73.2982 | 0.329495 |
| 77 | NM_000239, AI341872 | - | 146.61985 | 444.3954 | 0.329931 |
| 78 | X00437, BC030533, BC073930 | - | 47.69155 | 144.19535 | 0.330743 |
